# Differentiating Dynamic Cerebral Autoregulation Across Vascular Territories

**DOI:** 10.3389/fneur.2021.653167

**Published:** 2021-03-23

**Authors:** Navpreet Reehal, Stephanie Cummings, Michael T. Mullen, Wesley B. Baker, David Kung, William Tackett, Christopher G. Favilla

**Affiliations:** ^1^Department of Neurology, University of Pennsylvania, Philadelphia, PA, United States; ^2^Department of Neurology, Children's Hospital of Philadelphia, Philadelphia, PA, United States; ^3^Department of Neurosurgery, University of Pennsylvania, Philadelphia, PA, United States

**Keywords:** cerebral autoregulation, cerebral blood flow, cerebral hemodynamics, transcranial Doppler, transfer function analysis

## Abstract

**Objective:** Transcranial Doppler is commonly used to calculate cerebral autoregulation, but measurements are typically restricted to a single cerebral artery. In exploring topographic heterogeneity, this study reports the first thorough comparison of autoregulation in all major cerebral vessels.

**Methods:** In forty healthy adults, flow velocity was monitored in the anterior, middle, and posterior cerebral arteries, and synchronized with arterial blood pressure. A transfer function analysis provided characteristics of autoregulation by quantifying the relationship between blood pressure and cerebral blood flow velocity.

**Results:** Phase, which quantifies the time course of autoregulation, was similar in all vessels. Gain, which quantifies the magnitude of hemodynamic regulation, was lower in posterior cerebral artery, indicative of tighter regulation. However, after adjusting for baseline flow differences in each vascular territory, *normalized* gain was similar in all vessels.

**Conclusions:** Discriminating dynamic cerebral autoregulation between cerebrovascular territories is feasible with a transcranial doppler based approach. In the posterior cerebral artery of healthy volunteers, *absolute* flow is more tightly regulated, but *relative* flow regulation is consistent across cerebrovascular territories.

**Significance:** The methodology can be applied to focal disease states such as stroke or posterior reversible encephalopathy syndrome, in which the topographic distribution of autoregulation may be particularly critical.

## Introduction

Cerebral autoregulation (CA) describes the ability to maintain stable cerebral blood flow (CBF) despite fluctuations in blood pressure (BP), thus protecting the brain from hypoperfusion and hyperperfusion (1, 2). In acute stroke, subarachnoid hemorrhage, or traumatic brain injury, impaired CA contributes to secondary brain injury (3–5). CA impairment is also associated with cerebral small vessel disease and dementia (6, 7). In patients with carotid stenosis, CA impairment predicts stroke risk and cognitive decline (8, 9). A reliable approach to CA quantification is critical to understanding the pathophysiology of multiple disease states, and holds the potential to personalize care.

CA is typically assessed in a single cerebral vessel, neglecting potential topographic heterogeneity. There are many differences between the anterior and posterior circulation, including absolute CBF (10), vascular tone (11), autonomic innervation (12), and metabolism (13). Thus, CA may vary across vascular territories, both in healthy individuals and in various disease states (14–16). Quantifying CA in different territories could improve our understanding of the pathophysiology underlying cerebrovascular disease. In focal diseases, such as stroke or posterior reversible encephalopathy syndrome (PRES), the topographic distribution of CA may be particularly critical.

Dynamic CA (dCA) is a well-studied method for quantifying and reporting autoregulatory function. By synchronizing waveforms from transcranial Doppler ultrasonography (TCD) and arterial BP, a transfer function analysis (TFA) uses a Fourier decomposition of the two waveforms to quantify the effect of spontaneous BP fluctuations on CBF (17, 18). This approach negates the need to induce a BP change with a maneuver or medication and quantifies both the dampening effect and speed of CA. Typically, TCD assessments of dCA rely on middle cerebral artery (MCA) measurements (19); however, TCD reliably differentiates intracranial vessels, thus facilitating dCA measurements in other vessels. Still, a thorough comparison of the anterior (ACA), middle (MCA), and posterior (PCA) cerebral arteries is lacking.

Thus, the current study aimed to calculate dCA parameters in the ACA, MCA, and PCA in healthy volunteers, to test the hypothesis that there are differences in dCA between the anterior circulation (ACA and MCA) and posterior circulation (PCA).

## Materials and Methods

### Subjects

Forty healthy adult volunteers were enrolled in this study at the Hospital of the University of Pennsylvania between 6/21/19 and 8/8/19. Individuals were eligible if they were at least 18 years but excluded if they had a history of stroke, structural brain lesion, cervico-cerebral vascular abnormality, skull defect, or prior cranial surgery that would interfere with TCD monitoring. The protocol was approved by the University of Pennsylvania Institutional Review Board (protocol #833083). The study was conducted according to the principles expressed in the Declaration of Helsinki. Written informed consent was signed by each study participant prior to enrollment.

### Hemodynamic Monitoring

Subjects were positioned in the supine position, with head-of-bed elevated to 30°. Bed position may impact cerebral hemodynamics, so the bed position was held constant for all measurements and all subjects. The room was quiet and temperature controlled (22°C). The same room and bed were used for all subjects. Cerebral blood flow velocity (CBFv) was assessed using a Spencer Technologies ST3 TCD. A 2 MHz ultrasound probe was positioned over the subject's temporal bone window and adjusted to identify the ACA, MCA, and PCA. Each vessel was confirmed using standard velocity ranges, depth, and probe positioning (20). The ultrasound probe was secured to the subjects head using a Spencer Technologies Marc 600 transducer fixation headframe. Each vessel was sequentially monitored for 5 minutes. Symmetry was assumed in this healthy population (21), so data were collected from the right hemisphere to consolidate the protocol.

A finger plethysmograph system (Finometer® Pro, Finapres Medical Systems) was secured to the wrist and third digit was used to provide a continuous non-invasive measurement of the arterial blood pressure waveform. An inflatable brachial cuff was placed on the same arm and used to calibrate the Finometer® Pro prior to data collection. Re-calibration was performed before TCD data collection for each cerebral vessel. CBFv and BP waveforms were digitized and time synchronized.

### Dynamic Cerebral Autoregulation Calculation

**Figure 1:**
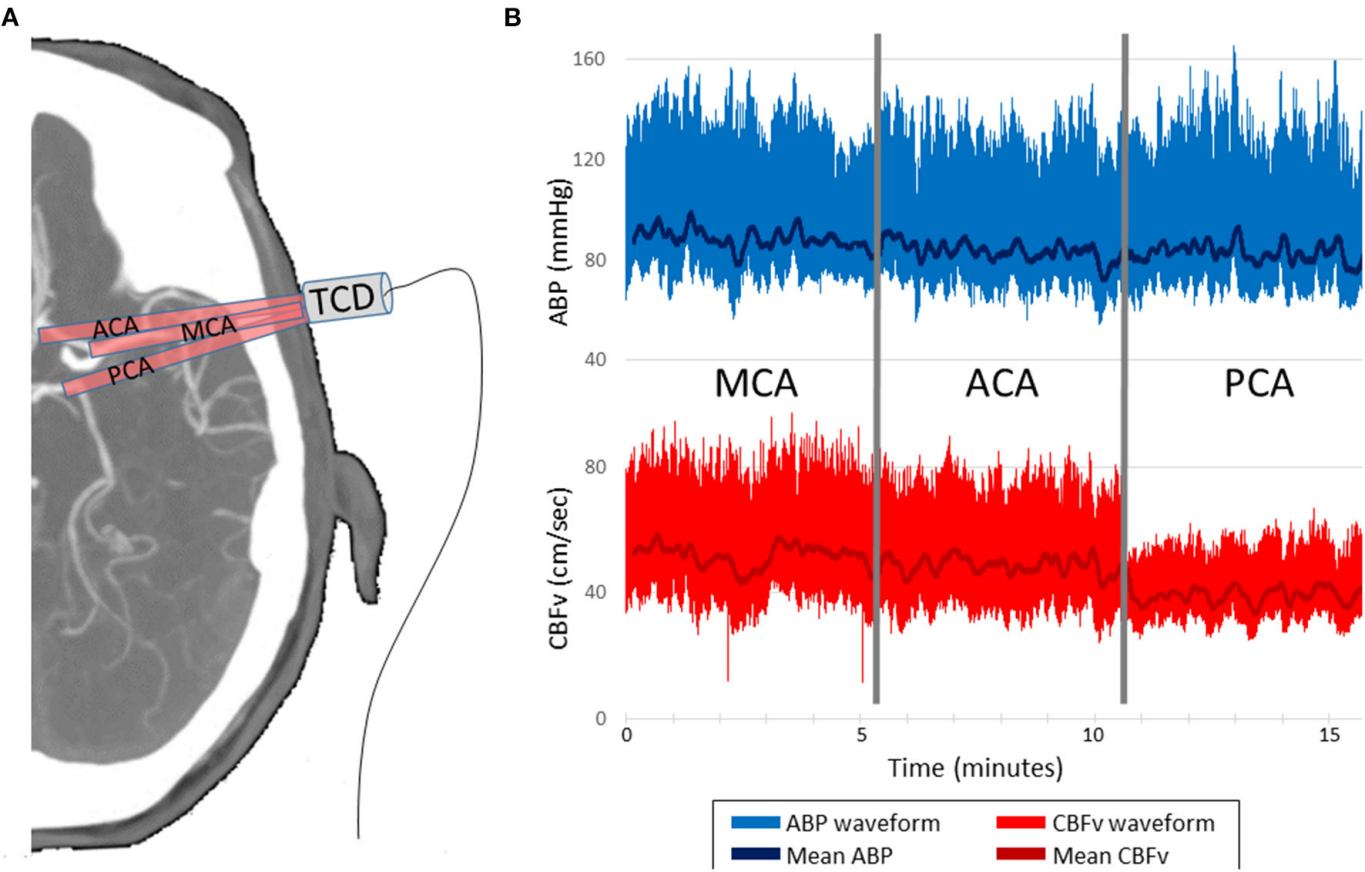
Representative data acquisition and time-series. **(A)** TCD probe is mounted over the temporal window in order to insonate the ACA, MCA and PCA, 5 min per vessel. **(B)** CBFv is synchronized with ABP, which serves as the input for the transfer function analysis. CBFv and ABP waveforms and mean values are depicted. TCD indicates transcranial Doppler. ABP indicates finometer measured arterial blood pressure. CBFv indicates TCD measured cerebral blood flow velocity. ACA indicates anterior cerebral artery. MCA indicates middle cerebral artery. PCA indicates posterior cerebral artery.

To calculate dCA, TFA quantifies the relationship between the input signal, the arterial BP waveform, and the output signal, the CBFv waveform. Exemplar BP and CBFv waveform data from the ACA, MCA, and PCA vessels is shown in Figure 1. TFA was performed using a Matlab script and algorithm provided by the International Cerebral Autoregulation Research Network (CARNet: www.car-net.org), to calculate gain, normalized gain, phase, and coherence for each vessel across three frequency bands (very low frequency (VLF): 0.02 – 0.07 Hz; low frequency (LF): 0.07 – 0.2 Hz; high frequency (HF): 0.2–0.5 Hz) (18). Gain (cm.s^−1^.mmHg^−1^) quantifies the damping effect of autoregulation (lower gain indicates more effective CA). Normalized gain (%.mmHg^−1^) accounts for mean CBFv differences between ACA, MCA, and PCA by using relative changes in CBFv rather than absolute. Phase, calculated in degrees, quantifies the time delay of cerebrovascular adaptation (larger phase shift indicates more effective CA). The coherence function assesses the validity of phase and gain estimates at each frequency band.

TCD and BP data were sampled at 200Hz. During data collection, care was taken to ensure the TCD signal was strong and largely free of artifact. If the vessel insonation was interrupted, the monitoring was extended to ensure 5 minutes of artifact-free monitoring. Data are synchronized during collection, so re-alignment was not performed during post-processing. After collection, raw waveform data were manually inspected to ensure they were free of excessive noise and artifact. Data from one subject was discarded due to excessive noise and low quality TCD data. For all other subjects, because of the lack of substantial artifacts, the waveform data were analyzed and used for the TFA, rather than beat-to-beat data. Low or high-pass filtering was not performed. A 100-second window length with 50% overlap was applied during TFA. The coherence threshold is inversely proportional to the duration of data collection. Based on 5 minutes of data collection for each vessel, a coherence threshold of 0.29 was applied, which represents the 95% confidence limit based on Monte Carlo simulation (18). If a coherence value was below the standard threshold, phase and gain values were discarded, as per the CARNet algorithm. As a result, the final analyzable sample size varied across frequency bands and vessels (Table 1).

**Table 1:** Hemodynamic data and TFA results.

	**ACA**	**MCA**	**PCA**	***P*-value**
Mean flow velocity, cm.sec^−1^	39.8 (16.0)	50.6 (15.4)	27.5 (13.3)	<0.0001
Mean arterial pressure, mmHg	83.5 (14.7)	85.6 (10.7)	84.2 (16.4)	0.80
**VLF**	*n = 34*	*n = 33*	*n = 32*	
Gain	0.41 (0.15)	0.52 (0.26)	0.31 (0.11)	0.0001
Normalized gain	0.77 (0.28)	0.81 (0.33)	0.83 (0.27)	0.69
Phase	53.4° (32.0°)	57.9° (31.4°)	55.1° (28.0°)	0.84
Coherence	0.40 (0.16)	0.37 (0.15)	0.38 (0.18)	0.77
CBFv spectral power	10.8 (6.4)	10.1 (7.1)	12.3 (7.3)	0.30
BP spectral power	8.8 (6.3)	8.8 (5.2)	9.9 (5.5)	0.67
**LF**	*n = 39*	*n = 39*	*n = 36*	
Gain	0.52 (0.26)	0.59 (0.24)	0.37 (0.13)	0.001
Normalized gain	0.99 (0.37)	0.93 (0.37)	1.02 (0.38)	0.56
Phase	33.1 (24.3°)	34.2 (17.7 °)	30.9 (17.8°)	0.78
Coherence	0.47 (0.20)	0.48 (0.21)	0.48 (0.21)	0.90
CBFv spectral power	9.4 (5.3)	8.3 (4.8)	8.7 (4.1)	0.66
BP spectral power	7.2 (5.1)	6.4 (3.8)	7.4 (5.3)	0.97
**HF**	*n = 38*	*n = 39*	*n = 35*	
Gain	0.57 (0.33)	0.61 (0.32)	0.44 (0.19)	0.03
Normalized gain	1.1 (0.53)	0.94 (0.44)	1.16 (0.55)	0.17
Phase	−2.7 (17. 3°)	3.0 (21.7°)	−8.6 (23.5°)	0.06
Coherence	0.34 (0.16)	0.40 (0.23)	0.31 (0.17)	0.15
CBFv spectral power	3.9 (2.4)	3.8 (3.2)	4.2 (2.1)	0.16
BP spectral power	2.2 (1.7)	2.7 (2.3)	2.0 (1.6)	0.53

*Gain is presented as cm.s^−1^.mmHg^−1^*.

*Normalized Gain is presented as %.mmHg^−1^*.

*Phase is presented as °*.

*BP and CBFv spectral power are presented as (mmHg)^2^ and (cm/s)^2^, respectively*.

*P-values were calculated by repeated measures one-way ANOVA*.

*CBFv indicates cerebral blood flow velocity*.

*BP indicates blood pressure*.

*Sample sizes vary because data were discarded if there was a failure to meet the standard coherence threshold for each frequency band in each vessel*.

Finally, for a secondary analysis, the CARNet algorithm was used to compute the spectral powers of CBFv and BP across frequency bands and vessels for the analyzable sample size. Low frequency power in hemodynamic signals have been proposed as biomarkers of neuronal activity (22, 23).

### Statistical Analyses

Summary statistics are presented using means and standard deviations. For all statistical tests, a p-value of < 0.05 was deemed to represent statistical significance. Phase, gain, and normalized gain values were tested for normality using Shapiro-Wilk normality test. The primary goal was to assess whether dCA varied by vascular territory. To this end, we compared phase, gain, and normalized gain between the ACA, MCA, and PCA vessels with repeated measures ANOVA, and if significance was found, a paired t-test between each group was used (i.e., ACA vs MCA, ACA vs PCA, MCA vs PCA). Analyses were repeated for each frequency band. In a secondary analysis, the same procedure was used to compare low frequency spectral powers of CBFv and BP between the ACA, MCA, and PCA vessels. All statistical analyses were performed in STATA/SE version 15.1 (StataCorp LLC, College Station, TX).

## Results

Forty consecutive healthy volunteers completed the study protocol. The median age was 21 years (IQR: 20–29) and 68% were female. Subject reported race was 60% Caucasian, 18% Asian, 12% African American, and 10% Other. Vascular risk factors were very uncommon: 8% of subjects had well controlled hypertension, and no subjects had a history of stroke, diabetes, hyperlipidemia, coronary artery disease, or heart failure. The monitoring protocol was well tolerated. Three subjects (8%) reported transient discomfort due to the TCD headframe, which resolved at the completion of the protocol and removal of headframe. No subjects elected to interrupt or terminate the protocol prior to completion. Data from the PCA in one subject was technically limited (waveform poorly captured) and was thus discarded. Data from all other vessels was analyzed.

**Figure 2:**
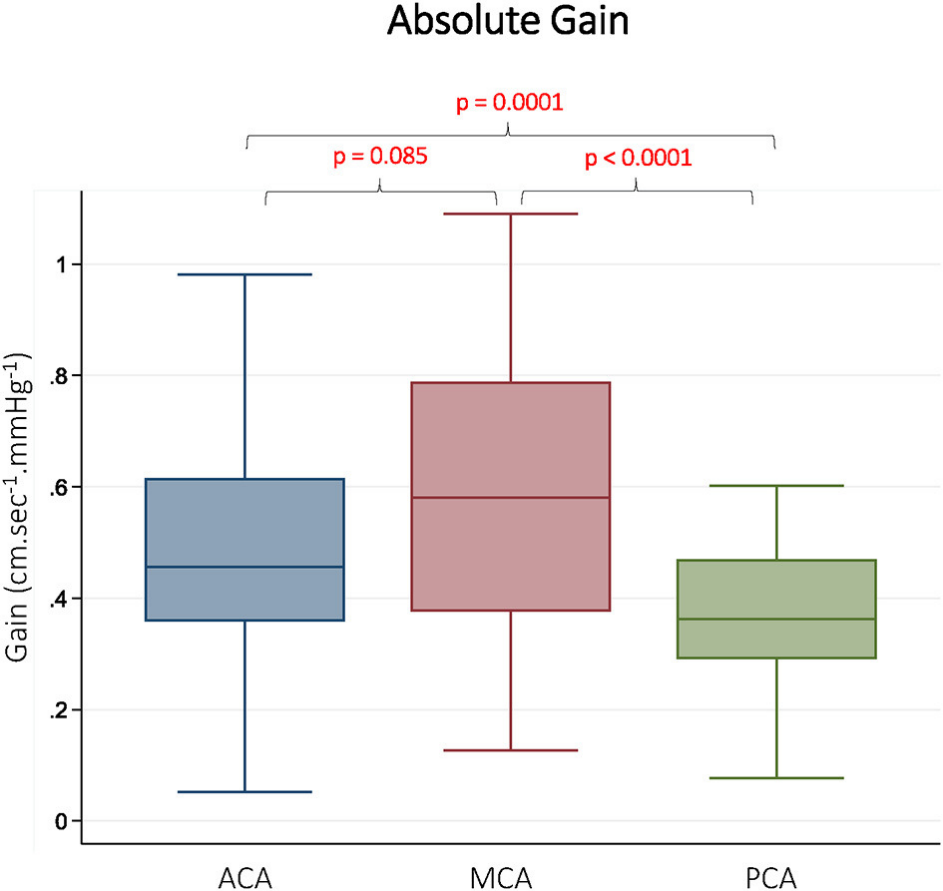
Absolute Gain in Different Vascular Territories. Gain is presented from the low frequency band (0.07–0.2 Hz). ACA indicates anterior cerebral artery. MCA indicates middle cerebral artery. PCA indicates posterior cerebral artery. Reported *p*-values were calculated by paired *t*-tests.

**Figure 3:**
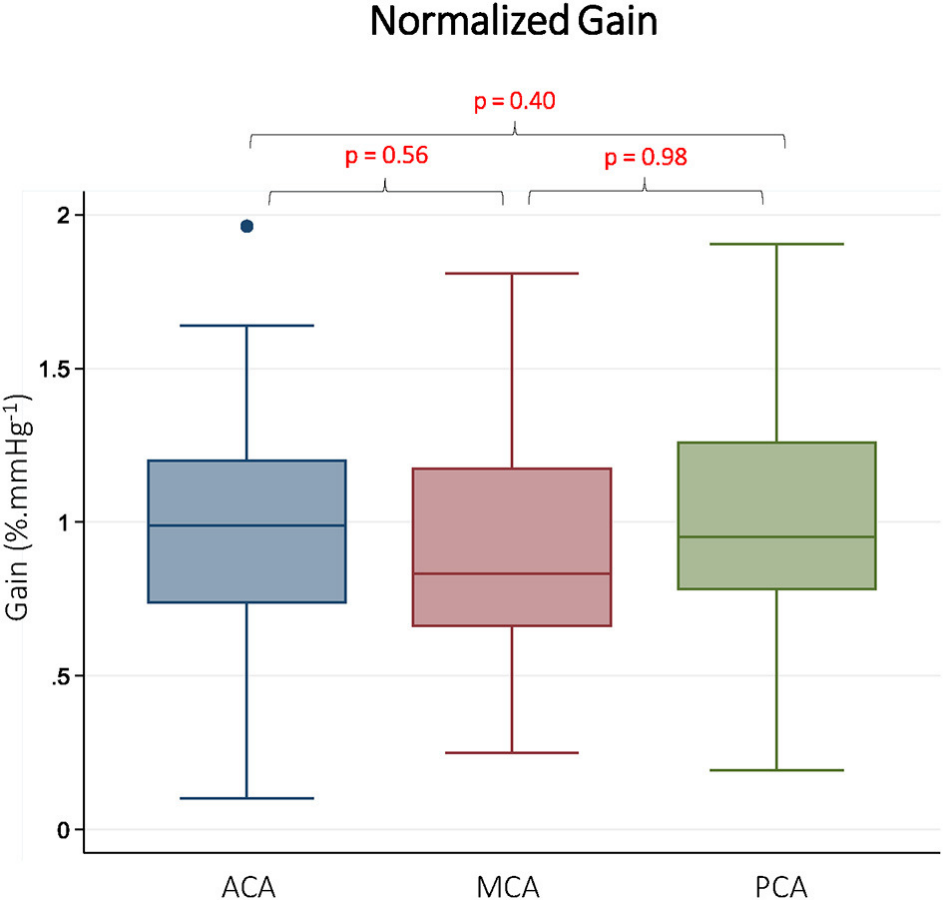
Normalized gain in Different Vascular Territories. Normalized gain is presented from the low frequency band (0.07–0.2 Hz). ACA indicates anterior cerebral artery. MCA indicates middle cerebral artery. PCA indicates posterior cerebral artery. Reported *p*-values were calculated by paired *t*-tests.

CBFv and BP summary data, along with TFA results are summarized in Table 1. As expected, there were differences in mean CBFv between vessels. BP was stable between measurements performed on each vessel, and <10% of gain and phase values were discarded due to inadequate coherence. The resulting samples for each vessel and frequency band are reported in Table 1. The gain was different between vessels in all 3 frequency bands. In pairwise comparisons, gain was similar in the ACA and MCA, but significantly lower in the PCA. Representative data from the LF band are presented in Figure 2. Normalized gain, which accounts for differences in absolute CBFv, was similar across vessels (Figure 3). No difference between vessels was observed with respect to phase or coherence. Similarly, CBFv and BP spectral power was similar in each vessel (Table 1). These findings were consistent for all frequency bands.

## Discussion

In healthy volunteers, phase was similar in all cerebral vessels, indicating a consistent timing of the autoregulatory response. Lower gain in the posterior circulation raises the possibility of more tightly regulated CBF in this territory. However, after normalizing for differences in CBFv between vessels, this difference was no longer observed. While prior comparisons have been made between MCA and PCA in healthy and disease states (14, 15, 24–26), this study represents the first comparison across the ACA, MCA and PCA. Further, previous work comparing MCA and PCA dCA often fails to report both gain and normalized gain across all frequency bands. The methodology we describe represents a thorough and easily reproducible protocol that can be used to quantify possible topographic heterogeneity in focal disease states such as stroke, subarachnoid hemorrhage, and PRES. Similarly, focal variant anatomy could be addressed in future work.

### Anterior and Posterior Circulation

A difference between anterior and posterior circulation autoregulation is physiologically plausible, as these two territories receive different sources of flow (carotid vs vertebrobasilar system) and varying degrees of autonomic innervation (12). Results of prior studies have, however, been inconsistent. A study of young adults revealed similar MCA and PCA autoregulation (26), but greater autoregulatory properties have been reported in the cerebellar vasculature (16). It is unclear if this highlights a difference between anterior and posterior hemodynamics, or supratentorial and infratentorial hemodynamics. Rosengarten et al. observed more rapid regulation in the posterior circulation and smaller absolute changes in the posterior circulation, but this may be related to differences in baseline CBFv (14). On the other hand, Haubrich et al. observed higher gain in the PCA as compared to MCA in older adults (25). Age is an important consideration, as PCA gain may disproportionately increase with age (15). In the current study, very few older subjects were included, thus precluding a secondary age-based analysis.

### Absolute and Normalized Gain

It is unclear if absolute gain (cm.s^−1^.mmHg^−1^) or normalized gain (%. mmHg^−1^) is more informative of autoregulatory function. Normalization is an appealing concept when comparing data from multiple subjects or multiple vessels with varying CBFv, but absolute changes in CBFv may also be physiologically relevant. For example, age has been observed to impact MCA absolute gain, but not normalized gain (27). On the other hand, patients with atrial fibrillation have higher normalized gain (LF band) than hypertensive patients, but this discrepancy is not seen in absolute gain (28). Absolute gain and normalized gain have diverging responses to changes in carbon dioxide (29). This is not to say that absolute gain is more informative than normalized gain, but rather that exclusive reporting of only relative or absolute gain may yield misleading results, thus highlighting the need for complete reporting of TFA results (18). Discrepancies may be particularly relevant in disease states, such as stroke, in which there may be large differences in CBFv.

### Transfer Function Analysis

The TFA was performed over three standard frequency bands (VLF, LF, and HF). Trends in gain and phase were consistent across frequency bands. Spontaneous BP oscillations in the VLF and LF range are more likely driven by autonomic vascular tone, less confounded by the cardiac or respiratory cycle (30). Many investigators therefore focus on LF and/or VLF bands, but this leads to inconsistent reporting of dCA result in the literature (31). With increasing frequency, BP oscillations may also be modulated by cardiac and respiratory variables, thus complicating the interpretation of the BP-CBF relationship. Complete reporting of frequency bands, regardless of the interpretation, is critical to transparency and reproducibility (18). To that end, this study utilized a standardized TFA script and algorithm that is available through CARNet (www.car-net.org).

Finally, low-frequency spectral powers of hemodynamic signals have been proposed as biomarkers of neurovascular coupling and neural activity (22, 23). Originally developed for functional magnetic resonance imaging (fMRI) (23), a more recent paper used TCD to measure low-frequency power in CBFv signals in post cardiac arrest patients, in which a difference in spectral power was observed between survivors and non-survivors (32). The measured low-frequency spectral powers of hemodynamic signals reported herein in Table 1 can be used to power future studies involving these biomarkers.

### Limitations

There are limitations to this study. TCD data was collected unilaterally to optimize quality, assuming symmetry in this young, healthy cohort. Bilateral comparison could be particularly important in older subjects or focal disease states. TCD also provides a measure of CBFv rather than CBF. These two terms are proportional assuming the vessel trunk remains stable, which is a reasonable assumption during a brief study conducted at rest. The young age of the cohort limits the external validity when considering subjects of older age or with vascular risk factors. However, the objective was not to draw broad conclusions, but rather to report results for young, healthy adults and highlight a reproducible methodology that can easily be applied to additional populations in future work. A range of ages could be explored in a future healthy volunteer study before applying this protocol to older patient populations. Partial pressure of CO_2_ impacts vascular tone and CBF, so it may confound our results, though it was assumed that during the monitoring period (15 min at rest), CO_2_ remained stable, which has been the case in multiple prior studies (33). End-tidal CO_2_ could be monitored in future work to ensure there is no confounding. Variant anatomy (e.g., fetal PCA) may impact hemodynamics, but cannot be accounted for in the current study because subjects do not have vessel imaging for review. Variant anatomy could be specifically considered in future work. The TFA methodology is somewhat limited by the assumption that CA is a linear control system. Nonetheless, it is a well-accepted approach to CA quantification.

## Conclusion

Discriminating dynamic cerebral autoregulation between cerebrovascular territories is feasible with a TCD-based approach. In healthy young adults, the time delay of autoregulation is consistent across territories, but the magnitude of hemodynamic regulation is more tightly regulated in the posterior circulation. Importantly, typical flow velocities are lower in the posterior circulation, so when considering percent hemodynamic changes, rather than absolute, no differences are observed between territories. Both absolute and normalized metrics contribute to the understanding of cerebrovascular physiology. The methodology described here can be easily reproduced and deployed to explore topographic characteristics of autoregulation in multiple disease states, in particular focal disease such as stroke.

## Data Availability Statement

The raw data supporting the conclusions of this article will be made available by the authors, without undue reservation.

## Ethics Statement

The studies involving human participants were reviewed and approved by the University of Pennsylvania Institutional Review Board. The patients/participants provided their written informed consent to participate in this study.

## Author Contributions

NR performed data collection, and assisted in the data analysis, and contributed to manuscript preparation. SC assisted with data collection and contributed to manuscript preparation. MM assisted with study design, assisted with analysis, and contributed to manuscript preparation. WB assisted with data analysis and contributed to manuscript preparation. DK assisted with study design and contributed to manuscript preparation. WT assisted with data analysis and contributed to manuscript preparation. CF provided overall study design, assisted with data collection, performed primary analysis, and contributed to manuscript preparation. All authors contributed to the article and approved the submitted version.

## Conflict of Interest

The authors declare that the research was conducted in the absence of any commercial or financial relationships that could be construed as a potential conflict of interest.
